# Self-reported use of internet by cervical cancer clients in two National Referral Hospitals in Kenya

**DOI:** 10.1186/1756-0500-5-559

**Published:** 2012-10-09

**Authors:** Lucy W Kivuti-Bitok, Geoff McDonnell, Ganesh P Pokhariyal, Abdul V Roudsari

**Affiliations:** 1 School of Nursing Sciences, University of Nairobi, P.O Box 19676-KNH-00202, Nairobi, Kenya; 2 Centre for Health Informatics, University of New South Wales, Sydney, NSW, 2052, Kenya; 3 School of Mathematics, University of Nairobi, P.O Box 30197–00100, Nairobi, Kenya; 4 School of Health Information Science, University of Victoria, PO Box STN CSC, Victoria, British Coloumbia, V8W 3P5, Canada

**Keywords:** Internet use, Cervical cancer, E-health, Mobile phones, Kenya

## Abstract

**Background:**

Cervical cancer remains a devastating disease in Kenya accounting for more than 2000 deaths each year. Lack of information on cervical cancer prevention and management has been attributed to the apathy among women in seeking health interventions. Use of internet-based and mobile e-health tools could increase information access among cervical cancer patients. The objective of the study was; to establish the extent of use of mobile phones and internet by cervical cancer patients in accessing information related to cancer treatment and management.; find out the characteristics of patients associated with internet use and identify barriers faced by the patients in internet use. A cross sectional descriptive survey of 199 cervical patients visiting the two main referral hospitals in Kenya was done. A structured questionnaire was used to collect data.

**Findings:**

The average length of illness was 2.43 years (SD ± 3.0). Only 7.5 %( n=15) reported to having used the internet as a source of information. 92.5 %( n=184) did not use internet. With Multiple options, 70.9% did not know how to use a computer, 29.2% did not have access to a computer, 14.6% lacked the money to use computers at the local cyber cafe while other barriers identified accounted for 11.1%. Patients reported that the internet had an important role in the management of cancer of the cervix in health education (17.6%), online consultation (14.6%), booking of patients (13.6%), referrals (8.5%) and collecting data (7%). The 96.5% of the respondents who had access to a mobile phone, recommended mobile phones for health education messages (31.7%), reminder alerts for medication (29.7%) and booking appointments (21.6%). There was a statistically significant association between income of the patients and internet use (p = 0.026) in this study.

**Conclusions:**

There is low level use of the internet by cervical cancer clients attended in Public referral facilities in Kenya. This was attributed to; lack of knowledge on how to use computers and lack of access to a computer. High level of access to mobile phones was reported. This is an indicator of great potential for use of mobile phones in the management of cervical cancer through short messaging services (sms), without internet connectivity. There is even greater potential to internet use through web access via mobile phones.

## Introduction

Worldwide, Cervical cancer causes approximately 46,000 deaths each year in women aged 15–49 years and is estimated to account for 15% of all female cancers in developing countries [1]. In Kenya, cervical cancer continues to be a major health concern. Estimates indicate that every year 2635 women are diagnosed with cervical cancer and 2111 die from the disease [2]. It is also projected that in 2025, there will be 4074 new cases of cervical cancer in Kenya and that 3293 deaths will be as a result of cervical cancer [3]. Facility based studies have indicated as high as 10 – 15 new cases of cervical cancer in Nairobi each week [4]. Different measures have been employed in dealing with the rising cases of cervical cancer. These have included screening, vaccination, treatment and health education [5,6]. While access to information on cervical cancer management has contributed to reduction in cervical cancer in developed countries [7], it is different in developing countries. Lack of access to quality information on cervical cancer screening and management has been attributed to the low health status in developing countries [6,8]. It has been argued that investment in Information and Communications Technologies (ICT) in the health care sector has greatly improved health status in developed countries [9]. The World Health Organization(WHO) defines Electronic health (e-Health) as “ the cost-effective and secure use of information and communications technologies in support of health and health-related fields, including health-care services, health surveillance, health literature, health education, knowledge and research” [10]. In the Netherlands, for example, one study reported as high as 84% of direct and indirect internet use by cancer patients, while in the USA over 50% internet usage by cancer patients has been reported [11]. It is hoped that the same kind of investment in ICT could benefit developing countries. According to Odutola [9] developing countries are lagging behind in utilization of ICT in health care and hence may not realize the potential benefits of e-health. Development and investment of e-health in developing countries need to be contextual in nature in order to meet the peculiar characteristics of developing countries whereby; use of the mobile phone is the sole ICT communication gadget catching up drastically and the use of mobile phone in ehealth will be proportionally greater in low resource setting [12]. E-health can help improve access to healthcare in settings where traditional delivery of health care is affected by geographical barriers, high cost of transportation as well insufficient number of healthcare specialists, particularly for management of chronic conditions [10,12,13]. Benefits of internet use to patients include; more informed patients, ability of patients to seek second opinions, patients joining online communities for support, patients being able to communicate online with health care providers, supplementing information provided by health care providers and improving conceptualization of information gained, patients enjoy more privacy than face to face consultations [11,14-17]. The health care providers would benefit from decision making tools, time efficiency and cost containment [18]. Investment in e-health may result in overwhelming technological, financial and informational demands on the clients. Other competing interests in developing countries also need to be considered when determining investment priorities.

Use of internet for health management has been known to have possible detrimental effects on the patients. Even though many websites containing cancer information are available, much of the information contained in these websites may lack quality [16]. Lack of information on the other hand may increase anxiety among patients as well as hinder their health seeking behaviours [18,19]. It is important to study the characteristics of patients who use internet as a source of health information. Previous reports have established that factors such as level of education, age, sex, stage of progression of disease, computer access and economic status influence internet use among patients [8]. Identification of barriers to internet use by these patients is important in planning how to tackle the barriers in order to encourage use of ICT in cervical cancer management. Studies elsewhere have identified barriers related to users; including lack of computer skills, computer illiteracy, and inability to interpret the available information, slow internet connection and high subscription costs [20,21].

Few studies if any have been done in sub-Saharan Africa to determine levels of internet use among cancer patients. The objective of this study was to establish the extent of internet use by cervical cancer patients in seeking information related to cervical cancer; identify barriers faced by the patients while using internet and find out the characteristics of patients associated with internet use. The use of mobile phones by the patients was also explored.

## Findings

This short report is part of a larger study on clinical and socio-economic impact of use of e-health tools in cervical cancer management in Kenya. A descriptive cross sectional survey was done in the two main public referral hospitals in Kenya. Kenyatta National Hospital (KNH) and Moi Teaching and Referral Hospital (MTRH) were purposefully studied. KNH is the largest teaching and referral hospital in Kenya and is located in the capital city Nairobi and houses the sole radiotherapy facility in public sector. MTRH is the second largest teaching and referral hospital in Kenya and is located in Eldoret city in the rift valley province of Kenya. The two hospitals serve as the main referral hospitals for the country and receive over 70% of cervical cancer patients from all over the country. Data was collected between July 2011 and Dec 2011.

## Ethical considerations

Clearance was sought from the University of Nairobi/Kenyatta National Hospital (UON/KNH) and Ethics and Research Committee as well as the Institutional Research and Ethics Committee (IREC) of Moi University and Moi Teaching and Referral Hospital. Full disclosure to the participants was done. A written consent was obtained from the respondents. Confidentiality was assured as well as confirmation that participation in the study would not at all interfere with their management by the hospitals. No compensation was given to the respondents.

### Data collection

A pre-test was done in the Hospice at KNH in April 2011. These participants were not included as part of study subjects during data analysis. The pre-test ensured validity and reliability of the study tool. A purposive sample of all the cervical cancer clients attending oncology clinics and all the cervical cancer patients admitted in the wards was done. The term clients and patients has been used interchangeably in this document. The staff nurses identified the cervical cancer clients and introduced them to the research assistants. Of the two hundred and five (205) participants identified and approached, two did not consent while four were too sickly to participate. A structured questionnaire was then administered to clients and patients between July 2011 and December 2011. Three trained research assistants performed the data collection exercise. Data was analysed using Statistical Package for Social Scientist (SPSS) version 17.0.

### Socio demographic characteristics of patients presenting with cancer of the cervix at KNH and MTRH

A total of 199 female clients attending KNH (n = 129, 64.6%) and MTRH (n = 70, 35.4%) were identified and included in the study. The average duration since diagnosis with cancer of the cervix in the sample was 2.48 years (SD ± 3.0). The duration of illness ranged from one month to 24 years. The median duration of illness was 1.5 years (inter-quartile range 11 months to 5 years).

The socio demographic characteristics of the 199 female patients with cervical cancer at both study sites are summarized in Table 1 below. The mean age of participants was 47.8 ± 11.2 years, and the median age was 47 years (inter-quartile range 41 to 53 years). The percent distribution of patients by ten-year age groups is presented in Table 1. Most participants were middle-aged, with 42.2% (n = 84) of the patients in the age group 40–49 years.

**Table 1:** Socio-demographic characteristics of clients with cancer of the cervix

**Demographic characteristics**	**Frequency (n)**	**Percent (%)**
*Age category*		
20-29 years	9	4.5
30-39 years	28	14.1
40-49 years	84	42.2
50-59 years	43	21.6
60-69 years	19	9.6
70 + years	10	5.0
Not stated	6	3.0
*Highest level of education*		
No formal education	27	13.6
Pre-primary	16	8.0
Lower primary	25	12.6
Upper primary	81	40.7
O-level	36	18.1
A-level	5	2.5
Diploma	6	2.5
Degree	3	1.5
Masters degree	1	0.5
*Marital status*		
Single	25	12.6
Married	152	76.4
Separated	9	4.5
Divorced	1	.5
Other	12	6.0
*Occupation*		
House wife	63	31.7
Farmer	61	30.7
Business	22	11.1
Formal employment	11	5.5
Casual labor	6	3.0
Retired	6	3.0
Others	30	15.1

Mobile phone access was reported by 95.5 % (n=192) of the respondents.

The married patients (n=143) constituted 71.9% of the participants and represented the most common marital status followed by single patients comprising 12.6% (n=25) of patients. Approximately one third of the patients in this study reported that they were housewives (n = 63, 31.7%). A similar number of patients reported that their main occupation was farming (n = 61, 30.7%). The other commonly reported occupations were business and formal employment with 11.1% and 5.5% of all patients indicating they were engaged in these occupations respectively.

### Formal education

**Figure 1:**
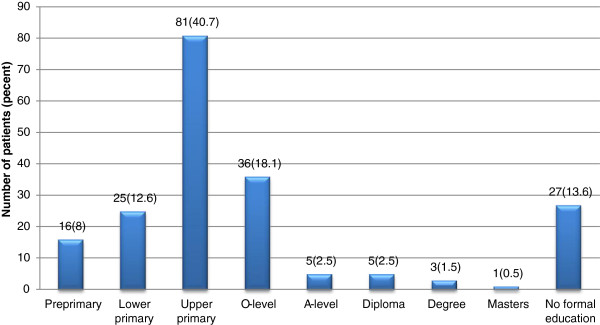
Level of Education of Cervical Cancer Clients.

A cumulative proportion of 86.4% of patients reported having had formal education. Conversely, 13.6% of patients in the study did not have any formal education (Figure 1). Most (40.7%) patients had completed upper primary education while 18.1% had post primary (ordinary level education).

When socio-economic status was examined, 65.3% (n=130) of patients were found to be in the lowest income category with monthly income below Kenya Shillings 10000(Approximately $150), and 11.1% (n=22) with income between Kenya Shillings 10000-50000 (Approximately $150 to $600) per month.

Table 2 shows that majority 93% (n=185) of the respondents sought information on cervical cancer form their doctors.

**Table 2:** Cited sources of information on Cervical Cancer

**Source of cancer information**	**Frequency**	**Percent**
Doctor	185	93.0
Nurse	72	36.2
Friend	36	18.1
Relatives	24	12.1
Radio	63	31.7
Television	47	23.6
Mobile phone	5	2.5
Billboard	7	3.5
Other source	5	2.5

This was followed by Nurses 36.2% (n-=72), the Radio as reported by 31.7% (n=63) while the T.V. accounted for 23.6% (n=47) as a source of information. Only 2.5% (n=5) of the patients used their mobile phones to source for information on cervical cancer despite 96.5% (n=192) of them having access to a mobile phone.

### Use of mobile phone in managing cervical cancer

It was noted that 96.5%( n=192) had access to a mobile phone .The respondents were asked to give their opinion on use of mobile phone in cancer management. The results were as shown in Table 3.

**Table 3:** Use of mobile phones by cervical cancer clients

**Use of mobile phones**	**Frequency**	**Percent**
***Number of reported uses***		
Participant did not state any use	86	43.2
Single use	72	36.2
At least 2 uses	41	20.6
**Specific uses Reported**		
Booking appointment	43	21.6
Reminder alerts for medication	59	29.7
Health education messages	63	31.7
Other use	4	2.0

### Patient self-reported use of internet in cancer diagnosis and treatment

Table 4 shows details of the patients’ use of internet with regards to their illness. Only fifteen (7.5%) of the patients in the study reported that they used the internet to get information on cancer of the cervix. The most common reasons for this low usage of the internet were lack of knowledge on how to use computers (70.9%) and lack of access to a computer (29.2%).

**Table 4:** Use of Internet by Cervical Cancer Clients and Reasons Given for Not Using Internet

	**Number (percent)**
Use internet to get information on cancer	
Yes	15 (7.5)
No	184 (92.5)
Reasons for not using internet	
Don’t know how to use computers	141 (70.9)
Lack access to a computer	58 (29.2)
Lack money to use a computer	29 (14.6)
Any other reason	22 (11.1)

Among the 15 patients who used the internet regularly to search information on cancer, three patients reported that they were directed by a nurse/ doctor and five reported that they used local search engines. Approximately half of these patients (n = 7) reported that they gained more knowledge about their disease or condition from using the internet.

**Figure 2:**
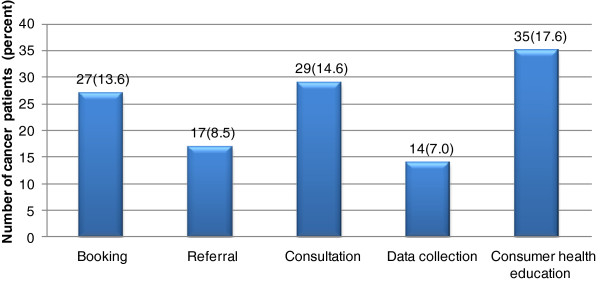
Use of mobile phone Technology as suggested by Cervical Cancer Clients.

The patients reported that internet had an important role in the management of cancer of the cervix. The different uses suggested by patients for IT in cancer treatments are shown in Figure 2 below.

The respondents were asked to identify functions they wished to be performed on the internet. Majority 17.6% (n=35) of the respondents preferred health education, this was closely followed by online consultation and booking. Less that 10% of the respondents chose referral and data collection functions.

### Association of income and internet use

There was a statistically significant association between income of the patients in this study and internet use (p = 0.026) as shown in Table 5. The highest rate of internet usage was among patients with incomes of between Kenya Shillings 10,000 to 50,000 per month (13.6%). The lowest rates of usage among patients who had some income were in the group of patients earning less than Kshs 10,000 a month (6.9%).

**Table 5:** Level of Income of Cervical Cancer Clients

**Income in KSHS**	**Internet use**		**Total**	**Fishers exact P value**
	**Yes**	**No**		
<10,000	9 (6.92)	121 (93.08)	130 (100)	0.026
10,000 to 50,000	3 (13.64)	19 (86.36)	22 (100)	
50,000 to 100000	1 (100)	0 (0)	1 (100)	
missing	2 (4.35)	44 (95.65)	46 (100)	
**Total**	**15 (7.54)**	**184 (92.46)**	**199 (100)**	

## Discussion

Access to quality information is paramount in the management of cervical cancer. The respondents in this study revealed different sources of cervical cancer information. These results supported studies done elsewhere which identified radio and television among the leading electronic sources of cancer information [6,22]. The internet was a less preferred source of information accounting for 38.8% in comparison to television and radio which accounted for 52% [22].This contradicted a study done earlier in Kenya which showed only 4.3% prevalence in the use of television and radio as sources of information on cervical cancer [6]. This trend may be attributed to the rising number of households with access to a television and radio in developing countries [23].

This study confirmed report by International Telecommunication Unions (ITU) that mobile phone access was higher than internet access in developing countries [24] and supports study by Patil [12] who reported that 64% of all mobile phone users are found in developing countries. The finding that 96.5% of the respondents had access to a mobile phone indicates a high level of cultural and social acceptance of mobile phones and high potential for their use [12,25]. Even though a high percentage of the respondents in this study had access to a mobile phone none of them reported access of internet from their mobile phones. It is possible that the cell phones offer very basic services and may not be internet enabled.

This study found a low level of self reported use of internet among cervical cancer patients in Kenya with only 7.1% of patients reporting use of internet in cervical cancer management. Few comparable studies if any have been done in developing countries. A study done in Malawi among health care professionals revealed that on 5.3% of respondents had access to internet facilities [26]. Other studies have been done in economic transitional nations. These are nations which have changed from traditional agricultural to industrial based economies and include; Malaysia, Singapore, South Korea Hong Kong, Indonesia and Thailand. Although the setting may not be comparable to developing countries, they may act as a guide in establishing the trends of internet use as a source of Health information. Noh et al. [22] found that in South Korea, 38.8% of cervical cancer patients aged less that 45 years used the internet as source of information. A study done in Malaysia [27] found that 22.5% of breast cancer patients used the internet for seeking information on health.

Studies in developed countries have reported internet access of 16-64% among cancer patients [28]. Generally it has been noted that technology accessibility is low in developing countries and rarely will a poor woman in a developing country have access to the internet [29]. Even when the poor woman in a developing country had access to internet, quality of the information accessed, poor connectivity, low capacity to interpret and utilize the information and decision making as well as cultural barriers may still be a hindrance to expected health benefits [28,29]. This study concurs with studies done elsewhere which identified lack of computer skills and lack of internet access as a barrier to use of ICT in healthcare [14] and that low income is associated with low use of technology [9,30].

The identification of potential functions of internet in management of cervical cancer is a good indicator of positive attitude towards e-health. This forms a good basis for developing a needs based e-health system and hence avoid the danger of pushing use of ICT down to the users [30].

## Conclusion

This study found that only a very small percentage of cervical cancer patients had access to the internet. The patients have a positive attitude towards use of internet in management of cervical cancer, hence acceptability and uptake of internet use is likely to be high. A large percentage of respondents had access to a mobile phone. This is an indicator of great potential for use of mobile phones in the management of cervical cancer through short messaging services (sms), where internet connectivity is low or unavailable. There is even greater potential to internet use through web access via mobile phones thus, a great promise in use of mobile technologies in management of cervical cancer.

### Limitations of the study

This study was done in public referral hospitals in Kenya and did not include data from Private institutions. Even though the two main private hospitals were approached to participate in this study, one declined as they indicated that they rarely received Cervical cancer clients and hence had insufficient numbers, while the other reported that they had too many research projects going on in the institution at the time and hence could not accommodate one more. The results can therefore not be generalized to the private institutions. Purposive sampling on the other hand has potential researcher bias. Generalization of these findings is therefore limited.

## Competing interests

The authors declare there are no competing interests.

## Authors’ contributions

L W.K. designed the study, analyzed the data and drafted the manuscript. G.M, G.P. P and A.R designed the study; and critically reviewed and revised the manuscript for important intellectual content. All authors read and approved the final manuscript.
